# Factors Influencing Trace Element Levels in the Blood of Tin Smelting Workers

**DOI:** 10.1097/JOM.0000000000002554

**Published:** 2022-06-08

**Authors:** Ludi Zhang, Boshen Wang, Huanxi Shen, Hengdong Zhang, Xin Liu, Lixin Zhong, Deye Liu, Dong Jiang, Yong Zhu, Baoli Zhu, Lei Han

**Affiliations:** From the Institute of Occupational Disease Prevention, Jiangsu Provincial Center for Disease Control and Prevention (Drs L. Zhang, Wang, H. Zhang, X. Liu, Zhong, D. Liu, Jiang, Y. Zhu, B. Zhu, and Han); and Key Laboratory of Environmental Medicine Engineering of Ministry of Education, School of Public Health, Southeast University (Drs L. Zhang, Wang, and B. Zhu), Nanjing; Kunshan Municipal Center for Disease Prevention and Control (Dr Shen); Center for Global Health, Nanjing Medical University (Dr B. Zhu); and Jiangsu Province Engineering Research Center of Public Health Emergency (Dr B. Zhu), China.

**Keywords:** trace element, blood, tin smelting workers, ICP-MS

## Abstract

This study is to assess the correlation between blood concentration ranges of eight elements of tin smelting workers from Guangxi Liuzhou and their job type, working years, age, and sex.

**Methods:**

We collected blood samples of 218 tin smelting workers from a Chinese tin smelting factory and determined the levels of elements by inductively coupled plasma mass spectrometry.

**Results:**

Within the blood concentrations of eight metal elements of the objects, the blood concentration of copper and zinc is affected by the job type of comprehensive work; that of arsenic and mercury is affected by refining; and that of chromium, cadmium, and lead is affected by primary smelting.

**Conclusions:**

We present the remarkable influence of four job types on the blood concentration of seven trace elements.

## Abstract 2

This research is of great significance to the prevention of occupational diseases for workers involved in tin. It can mainly reduce the occurrence of blood abnormalities in workers of tin smelting through occupational health protection for key positions.

The smelter processed various elements of tin smelting; the main product is the high-purity tin, and cadmium (Cd), lead (Pb), and so on are secondary products. The workers at the smelter were exposed to various substances, such as stannum (Sn), Pb, arsenic (As), Cd, dust, and other toxic materials.

Copper (Cu), zinc (Zn), As, Sn, and chromium (Cr) are essential nutrients required for numerous diverse biochemical reactions in the human body.^1^ Copper is the important component of metalloenzymes, including superoxide dismutase, cytochrome oxidase, and tyrosinase, with roles in hematopoiesis; as for Cu toxicity, neurodegenerative disorders such as Parkinson and Alzheimer diseases are supposed to be linked that Cu could contribute to their course or Cu toxicity.^2^ The Zn ion is a cofactor or activator of many enzymes, playing vital roles in vision, taste, and children's growth and development. People's exposure to Zn compounds that come from various sources such as food, water, air, and soil for a long time could generate toxic effects on the body, especially on the respiratory, digestive, and nerve systems, and it could also cause cancer,^3^ as it is a possible essential element and required for the synthesis of hemoglobin. Furthermore, As poisoning is a global health issue. Epidemiological studies showed that men's prolonged exposure to As could increase the risk of skin, lung, or bladder cancer.^4^ Studies have shown that As in the environment can induce reactive oxygen species (including hydrogen peroxide) to increase lung cancer risk.^5^ Stannum may be associated with protein biosynthesis. In the study of tin mine dust, it is found that lung epithelial cells are the primary target cells for mineral dust to induce cancer.^6^ Chromium is a cofactor of islet hormone and an essential component of pepsin and vitamin B_12_. The primary function of Cr is to regulate blood sugar metabolism. Excessive consumption of important trace elements, on the other hand, might be dangerous for health.^7^ Human health is harmed by Pb, mercury (Hg), and Cd. Lead compounds have been classified as carcinogens, neurotoxicants, and neurodevelopmental toxicants by the US Department of Health and Human Services and the Environmental Protection Agency,^8^ and new data on mechanisms of action provide biological plausibility for assessing Pb as a human carcinogen.^9^ In humans, Cd is a nonessential yet harmful element. Cadmium is ranked as the sixth most dangerous chemical to human health by the United Nations Environment Program. Intoxication with Cd causes renal failure as well as harm to the skeletal, hemopoietic, and cardiovascular systems.^10^ Mercury inhibits cell biological activity and proper metabolism by interfering with enzymes such as cytochrome oxidase, pyruvate kinase, and succinodehydrogenase. It also affects the central and vegetative nervous systems, the digestive tract, and other organs.^11^ Metals are absorbed through the lungs and the digestive system. Few metals, such as aluminum, may be eliminated by normal bodily functions, but others accumulate in the body and food chain, causing chronic effects. Exposure can come from a variety of places. Diet is the principal source of essential and “nonessential” trace elements for those who do not have occupational exposure. Ingestion or inhalation of particles may lead to exposure in places with high amounts of particular elements or in areas prone to pollution from industry.^12^ However, factors other than exposure, which are occasionally linked to it, may have an influence on element levels in people. Blood Pb was favorably related with testosterone and sex-hormone-binding globulin in Kresovich et al's^13^ study, whereas blood Cd was positively associated with sex-hormone-binding globulin. In Japanese children, there was a substantial negative connection between Pb and age. Lead levels were also much lower in Tokyo individuals than those in the other two areas.^12^

Biomonitoring of trace elements in human blood samples as exposure indicators has become an important technique in occupational and environmental medicine. Several countries have established methods for the analytical detection of elements in blood (e.g. Zn, Pb, Hg, Cd, etc.) and have developed reference value ranges.^14^ This report concerns the levels of a series of elements in the blood of tin smelting workers from a Chinese tin smelter in Guangxi province. The objectives were to present the concentration ranges of eight elements in the blood of the tin smelting workers by inductively coupled plasma mass spectrometry (ICP-MS) and to determine the influence of age, working years, sex, and job type on the blood levels of these trace elements. Therefore, the tin smelting workers were investigated.

## METHODS

### Studied Population

From a Chinese tin smelting factory in Guangxi Liuzhou, we selected 218 tin smelting workers for the sample. The smelter took processed raw ores as feedstock, and the primary process comprised raw material handling, crude smelting, feed mixing, sintering, and smelting to produce pure tin. The 218 tin smelting workers included all tin smelting workers who normally work for 3 consecutive months. Samples for element analysis were obtained from these 218 tin smelting workers. All participants were asked about their basic information, such as name, age, working years, and job type.

### Instrumentation

An Agilent 7500ce (Agilent Technologies Deutschland GmbH, Waldbronn, Germany) ICP mass spectrometer with a 27.12-MHz solid-state generator was used to examine the blood samples of these 218 tin smelting employees. An octupole-based reaction/collision cell with helium as a cell gas is used in the instrument. The cell was filled with a flow of 5.0-mL/min helium with a purity of 99.999% (V/V). The orifice diameters of the sampler and skimmer cones were 1.0 and 0.4 mm, respectively. A Babington nebulizer and a Scott spray chamber were used to introduce the samples. To ensure temperature stability and limit the amount of water vapor in the nebulizer gas flow, the spray chamber was Peltier cooled to 2°C. An injector tube with a large inner diameter of 2.5 mm is used in the ICP torch to reduce the danger of particle deposits or clogging. Table 1 shows the detection limit for eight elements in the blood.

**TABLE 1:** Limit of Detection for Eight Elements in the Blood

Elements	Blood, μg/L
^52^Cr	0.034
^63^Cu	0.033
^66^Zn	-*
^75^As	0.119
^114^Cd	0.009
^118^Sn	0.471
^201^Hg	0.115
^208^Pb	0.012

Abbreviations: As, arsenic; Cd, cadmium; Cr, chromium; Cu, copper; Hg, mercury; Pb, lead; Sn, stannum; Zn, zinc.

*0.1–1 ng/mL.

### Sample Preparation

Nine milliliters of blood was taken in lithium heparin monovettes designed for metal analysis (medical technology manufacturer in Nümbrecht, Germany). The first 9 mL of blood in the first monovette was always discarded to minimize contamination and to clean the needle of this device, and the blood in the second monovette was used for analysis. We leached 20 monovettes with deionized water and another 20 monovettes with 3% (V/V) nitric acid in ultrapure grade (Merck, Darmstadt, Germany) for 48 hours to look for contamination from the tube or the anticoagulant. All blood samples were maintained at −20°C after collection and tested within 2 days. The blood samples were brought to room temperature (20°C to 25°C) 2 hours before sample processing. They were gently mixed to homogenize the blood sample, and 500 μL of it was diluted with 100 L of 0.1% (V/V) Triton-X-100 solution (Sigma, St. Louis, MO) and 500 μL of the internal standard solution. A 5-mL bottle-top dispenser was used to fill a 10-mL autosampler polypropylene tube with a 0.5% (V/V) nitric acid solution of suprapure purity (pharmaceutical company in Darmstadt, Germany). Finally, the samples were homogenized using the React 2000 magnetic stirrer (Heidolph Instruments GmbH & Co KG, Schwabach, Germany).

### Calibration and Control Materials

The standard addition process was performed by diluting 500 μL of blood sample to 5-mL total volume for calibration. This external calibration with internal standards was converted from a standard addition calibration. Initially, multielement stock solutions for ICP-MS were made fresh in 100-L quartz glass flasks by dilution of 10-mg/L single-element standard solutions, taking into account the stability of elements in solution. In 10-mL polypropylene autosampler tubes, defined amounts of these stock solutions were added to a combination of 500-μL blood, 100-L 0.1% (V/V) Triton-X-100 solution, and 500-μL internal standard solution (pharmaceutical company in Darmstadt, Germany). Finally, 5 mL of 0.5% nitric acid was added to these solutions. Pipettes (volumes of 50 to 1000 L) and dispensers with changeable volumes from 1 to 5 mL are dilution devices (Eppendorf Corporate, Hamburg-Nord, Germany).

Each trace element's added concentrations in seven calibration solutions are 0, 0.0001, 0.001, 0.005, 0.010, and 0.100 mg/L, respectively. Zinc concentrations in the identical solutions are 0, 20, 50, 100, and 200 mg/L for the element. Before calibration, the elements' concentrations in all multielement calibration solutions were compared with two commercially available multielement calibration solutions (IMS-102 and IMS-103) from the same manufacturer. In all sample and calibration solutions, the internal standard concentration was 2.5-mg/L Tb. Daily, a 10-mg/L Rh solution was diluted in 2% (V/V) nitric acid to make the internal standard stock solution with 100-ng/mL Rh.

Seronorm's Trace Elements Whole Blood control levels 1 to 3 were used as internal quality control materials (laboratory equipment supplier in Billingstad, Norway). For each element, all control samples were examined at three distinct concentration levels. The commercial control materials were diluted according to the manufacturer's recommendations before being filled with Triton-X-100 solution, internal standard solution, and nitric acid solution in the same manner as the actual samples.

### Statistical Analysis

For the statistical study, we took into account sex, age, job type, and working years. The data were evaluated and contrasted based on the kind of variable: continuous versus categorical variables. The distribution of results from whole blood samples is frequently skewed. As a result, percentiles and geometric means were used to characterize the element concentrations. To calculate the correlation index and execute a multiple linear regression analysis, the nonnormally distributed values were transformed to a logarithmic form. For each of the four categories of labor, we created dummy variables. The concentrations of eight trace elements were used as a dependent variable, with age, working seniority, and job type as independent factors, in a multiple linear regression analysis of the subgroups. When the *P* values were less than 0.05, the results were considered significant. The statistical analysis was performed using the program SPSS® Advanced Statistical^TM^ 19.0 (SPSS Inc, Chicago, IL).

## RESULTS

**FIGURE 1:**
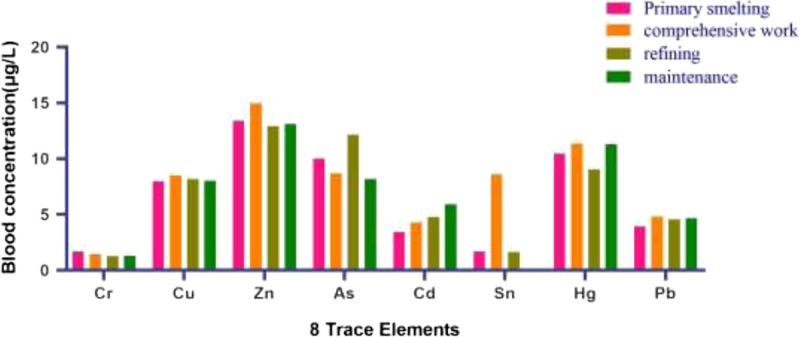
Blood concentration of the eight trace elements of workers in four positions.

Table 2 shows the information of the demographic characteristics of eight trace elements' concentration of the population divided by job type. This study investigated 218 tin smelting workers, including 190 men and 28 women; the correlation between the concentration of the eight elements of human blood and sex is summarized in Table 3, which shows the difference in blood Cd and Sn concentrations between men and women in this study was statistically significant, whereas the blood concentrations of the other six trace elements between men and women were not significantly different. The blood concentration of the eight trace elements among different work types is shown in Figure 1, and the data information in detail is shown in Table 4.

**TABLE 2:** Characteristics of the Subject by Job Type

Variables	Primary Smelting	Comprehensive Work	Refining	Maintenance
Population size	73	62	49	34
Sex, n (%)				
Men	64	49	43	33
Women	9	13	6	1
Age, yr				
Mean (SD)	35.6 (10.5)	43.84 (7.05)	36.2 (11.39)	40.29 (11.45)
Geometric average	34.07	43.19	34.38	38.63
Range	21–56	22–58	20–53	21–59
Median	34	45.5	35	39
Working years				
Mean (SD)	13.59 (10.62)	22.10 (8.26)	13.24 (10.92)	19.71 (12.58)
Geometric average	9.11	19.61	8.32	14.94
Range	2–38	2–36	2–3	3–41
Median	9	25.5	8	16.5

**TABLE 3:** Correlation Between the Eight Elements in the Blood

Elements	Male, Mean ± SD (Range), μg/L	Female, Mean ± SD (Range), μg/L	Significance
Cr	1.42 ± 0.64 (0.88–5.83)	1.49 ± 0.83 (0.85–4.13)	*P* = 0.824*
Cu	805.63 ± 131.41 (438.23–1285.93)	896.35 ± 158.08 (599.85–1317)	*P* = 0.325*
Zn	13639.48 ± 5328.37 (6672.30–45,391.18)	13962.07 ± 4994.49 (8189.93–30,832.18)	*P* = 0.917*
As	9.9 ± 5.67 (3.03–40.53)	9.28 ± 3.21 (3.13–18.65)	*P* = 0.2*
Cd	4.56 ± 3.82 (0.53–19.1)	2.95 ± 2.84 (0.85–12.95)	*P* < 0.05
Sn	28.79 ± 22.98 (12.75–74.88)	109.79 ± 80.9 (22.7–182.6)	*P* < 0.05
Hg	10.7 ± 5.58 (2.93–38.9)	9.31 ± 4.3 (2.9–19.9)	*P* = 0.334*
Pb	44.74 ± 18.11 (13.55–140.73)	41.47 ± 15.75 (18.27–75.63)	*P* = 0.976*

As, arsenic; Cd, cadmium; Cr, chromium; Cu, copper; Hg, mercury; Pb, lead; Sn, stannum; Zn, zinc.

*Not statistically significant.

**TABLE 4:** Blood Concentration of the Eight Trace Elements of the Studied Population by Job Type

Blood Concentration, μg/L	Primary Smelting	Comprehensive Work	Refining	Maintenance
Cr				
Mean (SD)	1.65 (0.86)	1.43 (0.68)	1.26 (0.48)	1.29 (0.36)
Geometric average	1.52	1.31	1.2	1.25
Range	0.9–5.83	0.85–4.13	0.88–3.35	0.88–2.2
Median	1.43	1.18	1.13	1.2
Cu				
Mean (SD)	798.319 (161.36)	849.31 (131.39)	817.58 (117.47)	799.19 (116.59)
Geometric average	782.53	839.54	809	791.4
Range	438.23–1285.93	535.88–1317	561.05–1088.47	629.65–1168.08
Median	774.58	836.18	838.53	783.96
Zn				
Mean (SD)	13,392.75 (4741.68)	14,948.55 (7311.45)	12,902.76 (3286.13)	13,109.47 (3882.9)
Geometric average	12,760.51	13,769.52	12,479.27	12,674.61
Range	7551.35–34,328.18	7756.85–45,391.18	6672.3–19,188.55	8751.28–29,210.43
Median	12,524.15	12,382.9	12,860.8	12,273.04
As				
Mean (SD)	10.03 (5.77)	8.65 (2.58)	12.16 (7.97)	8.18 (1.72)
Geometric average	8.91	8.3	10.28	8
Range	3.13–40.53	3.68–17.9	3.03–35.13	4.40–11.88
Median	8.75	8.34	9.96	8.31
Cd				
Mean (SD)	3.42 (3.64)	4.27 (3.30)	4.78 (4.13)	5.90 (3.69)
Geometric average	2.26	3.12	3.3	4.45
Range	0.53–18.73	0.63–12.95	0.68–19.1	0.6–14.88
Median	1.75	3.26	3.98	6.15
Sn				
Mean (SD)	16.77 (4.25)	85.58 (58.95)	16.39 (5.22)	
Geometric average	16.4	68.26	15.86	
Range	12.75–22.7	20.13–182.6	13.25–24.2	
Median	15.83	71.71	14.05	
Hg				
Mean (SD)	10.45 (6.36)	11.35 (4.99)	9.02 (4.73)	11.27 (4.92)
Geometric average	9.04	10.4	7.99	10.3
Range	2.9–38.9	3.90–32.90	2.93–24.6	4.58–22.43
Median	8.78	10.55	7.1	10.15
Pb				
Mean (SD)	39.15 (12.83)	48.02 (19.26)	45.65 (23.49)	46.74 (12.16)
Geometric average	37.1	44.74	41.01	45.29
Range	17.88–71.78	21.43–113.65	13.55–140.73	26.88–83.2
Median	37.8	41.7	42.43	44.18

As, arsenic; Cd, cadmium; Cr, chromium; Cu, copper; Hg, mercury; Pb, lead; Sn, stannum; Zn, zinc.

The parameters of general linear regression analysis for eight trace element concentrations in the blood from different job types are shown in Table 5, and the results show that the concentration of Cr in the blood of the research subject is only affected by the job type of primary smelting and the other three job types of comprehensive work have no effect on it and that age and working years of the subject also have no effect on the blood concentration of Cr. The blood concentration of Cu and Zn of the research subject is only affected by the job type of comprehensive work, and the other three job types of comprehensive work have no effect on it; age and working years of the subject also have no effect on the blood concentration of Cu and Zn. The blood concentration of As of the research subject is only affected by the job type of refining, and the other three job types of comprehensive work have no effect on it; the concentration of As decreases with age, but working years has no effect on the concentration of As. The concentration of Cd in the blood of the research subject is only affected by the job type of primary smelting, and the other three job types of comprehensive work have no effect on it; age and working years of the subject also have no effect on the concentration of Cd in the blood. The concentration of Sn in the blood of the research subject is only affected by the change of working age, and statistics show that the concentration of Sn increases with the increase of working age. The concentration of Hg in the blood of the research subject is only affected by the job type of refining, and the other three job types of comprehensive work have no effect on it; age and working years of the subject also have no effect on the concentration of Hg in the blood. The concentration of Pb in the blood of the research subject is only affected by the job type of primary smelting, and the other three job types of comprehensive work have no effect on it; age and working years of the subject also have no effect on the concentration of Cr in the blood.

**TABLE 5:** Parameters of General Linear Regression Analysis for the Blood Concentrations of Eight Trace Elements From Different Job Types

Independent Variables	Primary Smelting		Comprehensive Work		Refining		Maintenance		Working Years		Age	
	*t* (Beta)	*P*	*t* (Beta)	*P*	*t* (Beta)	*P*	*t* (Beta)	*P*	*t* (Beta)	*P*	*t* (Beta)	*P*
Cr	2.337 (0.309)	0.021*	1.029 (0.103)	0.306	−0.769 (0.076)	0.443	−0.326 (−0.031)	0.745	−0.242 (−0.022)	0.809	0.206 (0.019)	0.837
Cu	−0.533 (−0.040)	0.594	2.177 (44.757)	0.031*	0.803 (0.057)	0.423	0.804 (−0.018)	0.797	0.932 (0.065)	0.357	0.922 (0.065)	0.537
Zn	0.482 (0.036)	0.630	2.257 (1771.5)	0.025*	−0.443 (−0.03)	0.658	−0.085 (−0.006)	0.932	0.532 (0.037)	0.601	0.840 (0.059)	0.057
As	1.240 (0.09)	0.216	−0.278 (−0.02)	0.781	3.144 (2.708)	0.002*	−1.1423 (−0.076)	0.254	0.538 (0.098)	0.591	−2.509 (−0.166)	0.013*
Cd	−2.66 4 (−1.412)	0.008*	−1.56 (−0.117)	0.120	−0.007 (−0.102)	0.919	1.945 (0.136)	0.053	−1.396 (−0.095)	0.164	−0.969 (−0.066)	0.344
Sn	0.216 (0.051)	0.833	−0.21 (−0.123)	0.837	−0.117 (−0.026)	0.909			3.963 (3.170)	0.002*	0.111 (0.044)	0.914
Hg	−1.013 (−0.075)	0.312	0.721 (0.053)	0.472	−2.173 (−1.943)	0.031*	0.367 (0.026)	0.714	−0.403 (−0.028)	0.687	0.025 (0.002)	0.980
Pb	−3.097 (−7.770)	0.002*	0.654 (0.049)	0.514	−0.623 (−0.045)	−0.042*	−0.068 (−0.005)	0.946	0.338 (0.023)	0.736	0.429 (0.029)	0.668

As, arsenic; Cd, cadmium; Cr, chromium; Cu, copper; Hg, mercury; Pb, lead; Sn, stannum; Zn, zinc.

*Statistically significant.

## DISCUSSION

Tin ore contains many impurities, such as Pb, Zn, As, Cd, and so forth.^15^ Under the hot environment of the reverberatory furnace, tin oxide and oxide impurities are reduced to crude tin, producing dust and metallic oxides at the same time. Harmful smoke, which is not efficiently eliminated, would lead to occupational injuries, such as stannosis, pneumoconiosis, As poisoning, arsine poisoning, Cd poisoning, and skin lesion.^16^

This study showed the levels of eight elements in the blood of tin smelting workers from Guangxi Liuzhou. For all we know, this is the first time for a study to determine the concentrations of trace elements in the whole blood of tin smelting workers in China. The geometric average blood concentrations of the eight trace elements range from the Level of Detections (LODs) up to 32 μg/L for Cu and 518 μg/L for Zn. All trace element concentrations are in a large range for Cr (<0.034 to 0.233 μg/L), Cu (17.529 to 52.680 μg/L), Zn (266.892 to 1815.647 μg/L), As (<0.119 to 1.621 μg/L), Cd (0.021 to 0.764 μg/L), Sn (<0.471 to 7.304 μg/L), Hg (<0.115 to 1.556 μg/L), and Pb (0.542 to 5.629 μg/L). For some essential elements, there are significant differences from previous research. Compared with background levels for Cu (541 to 1475 μg/L) and Zn (2349 to 9492 μg/L) for blood samples of the general population in residents of a Beijing suburb, our geometric average concentrations for Cu (32.23 μg/L) and Zn (518.44 μg/L) are significantly lower.^17^ In addition, as for a study by Wang et al,^18^ the normal limits of 33 elements (Li, U, Be, Th, B, Pb, Mg, Tl, Al, Hg, Ca, Au, Ti, Ba, V, Cs, Cr, Sb, Mn, Cd, Fe, Ag, Co, Mo, Ni, Zr, Cu, Sr, Zn, Rb, Ga, Se, and As) in whole human blood were identified in the general population in a province in China. In this research, the average levels of BAs, BCr, BCu, and BZn were 5.99, 11.0, 838, and 11,126 μg/L, respectively. Compared with these data, the levels of BAs, BCr, BCu, and BZn in tin smelting workers were markedly lower than those in the healthy population of Hunan province. The main occupational hazard factors in the process of tin smelting are As hydride and arsenious oxides. Most of the As and its compounds produced in the process of sintering, roasting, and smelting in a blast furnace are received by the dust removal device. In these workplaces, especially the dust collection position, the skin and respiratory tract have access to high concentrations of As and its compounds. Arsenic slag and ashes would produce large amounts of As hydride gas when reacting with water, a major cause of As hydride occupational poisoning.^19^ As for the element Sn, the mean value was 4.2 μg/L in the control group in a study analyzing the content of 30 plasma elements in lung cancer women and control groups. According to the result, a significantly lower level of BSn (0.26 μg/L) was observed in the tin smelting workers in our study. Tin miners are mostly exposed to satanic oxide (SnO_2_) and partially tin sulfide, such as selenite ore (Cu_2_FeSnS and PbZnSn_2_). The main exposure substance of tin smelting workers is SnO_2_ dust. Tin smelting workers in a working environment with a smoke concentration of 9.362 mg/m^3^ have a risk of developing stenosis.^20^ Most patients have no chance of pulmonary function with rarely clinical symptoms, including cough, sputum, and chest tightness. The typical chest c-ray film is clusters of small round speckled shadows widely distributed in two lung fields. As for some elements with unknown biological function (Sn), the concentration of the actual sample is much lower than the LODs. Furthermore, the results of Sn should be explained discreetly. Geometric averages of blood concentrations of Pb, Hg, and Cd of the whole population were brought out by Wang et al^18^ in Hunan province with values of 61.4, 3.75, and 3.67 μg/L, respectively. Our geometric average concentrations for Pb, Hg, and Cd are 1.65, 0.35, and 0.12 μg/L, respectively, which are also lower compared with the values reported for Hunan province.

In our opinion, the dramatic differences of element concentrations in blood between tin smelting workers and healthy people not only attribute to dust exposure. Environmental background levels of trace elements in different areas also play a role and even cover the role of dust exposure. Unfortunately, we did not find the material about trace element concentrations in the blood of the healthy population from Guangxi Liuzhou, and this will be our further research.

In our study, the levels of BCr, BCu, BZn, BAs, BCd, BHg, and BPb were significantly associated with job type. In the section of primary smelting, BPb, BCd and BCr were the highest. BZn and BCu were the highest in comprehensive work. BAs and BHg was the highest in refining work. These date indicate that different job types are exposed to diverse hazard factors. BCu of women tends to be 15% to 17% higher than that of men.^21^ The results of the recent research showed that the higher concentrations of Cd were observed in men and those of Sn were observed in women (Table 4); combined with another study,^22^ in healthy non–occupational hazard exposure people, the blood concentration of Cd was higher in men compared with women, whereas tin was higher in women (*P* < 0.05). It has been hypothesized that estrogen-induced ceruloplasmin synthesis in the liver may lead to an increased Cu in blood.^23^ Tin smelters should pay attention to the monitoring of the concentration of Cr, Cd, and Pb elements in the workplace in the primary smelting position; As and Hg at the workplace of refining position; and Cu and Zn at the workplace of comprehensive work position. In addition, statistical analysis showed that the concentration of Sn in the blood of tin smelting workers in the factory increased with age; this may be caused by its accumulation in the human body, which should be known to the workers. It also showed that the concentration of As in the blood of the workers decreased with age. This may be due to the decrease in the absorption capacity of the human body for As elements with the increase of age, which needs to be further studied.

## CONCLUSIONS

The major aim of our research is the representation of blood concentration ranges of eight elements of the tin smelting workers in Guangxi Liuzhou in China by ICP-MS and the determination of the influence of job type, working years, age, and sex on the blood levels of these trace elements, because biomonitoring studies based on blood are rare in the literature in tin smelting workers of China. The application of ICP-MS allows fast and reliable measurement of different types of elements in the blood at the same time; this is especially true of trace elements (eg, Cu) and toxic metals (eg, Pb, Cd, Hg) that are essential for the human body. As for Sn, most concentrations of actual blood samples are lower than the LOD of ICP-MS. In addition, statistics showed that the blood concentration of the trace elements of tin smelting workers was affected by four different job types, the concentration of Sn increases with working age, and the concentration of As decreases with age. Concentrations in the range provided are meaningful for further research and toxicological studies.
